# Digital heart initiative: an ecosystem for digital discovery and precision medicine in cardiology

**DOI:** 10.1093/nsr/nwag302

**Published:** 2026-05-25

**Authors:** Haibo Ni, Chen Cao, You Zhou, Yu Bai, Jingyi Sheng, Ying Wang, Ning Gu

**Affiliations:** Medical School, Nanjing University, China; Department of Cardiology, Cardiovascular Disease Center, Institute of Clinical Medicine, Jiangsu Key Laboratory for Cardiovascular Information and Health Engineering Medicine, Nanjing Drum Tower Hospital, Medical School, Nanjing University, China; School of Biomedical Engineering and Informatics, Nanjing Medical University, China; Medical School, Nanjing University, China; Medical School, Nanjing University, China; School of Biological Science and Medical Engineering, Southeast University, China; School of Biological Science and Medical Engineering, Southeast University, China; Medical School, Nanjing University, China; Department of Cardiology, Cardiovascular Disease Center, Institute of Clinical Medicine, Jiangsu Key Laboratory for Cardiovascular Information and Health Engineering Medicine, Nanjing Drum Tower Hospital, Medical School, Nanjing University, China

## Abstract

This paper proposes the development of a digital heart ecosystem comprising four interconnected micro-ecosystems: devices and data; digital tools; digital discovery and precision cardiology; and infrastructure and governance.

Cardiovascular diseases remain the leading cause of mortality [1]. Clinical advances have been hampered by the complexity of the cardiovascular system, a network of biophysical machineries coupling energetics with electrical, mechanical, and fluid workloads while interacting with immune processes and other organs across multiple spatiotemporal scales. Innovating cardiovascular therapeutics thus requires untangling this multiscale complexity and leveraging its information flows, motivating a paradigm shift to digitalize cardiovascular pathophysiology for patient-specific medicine.

Digital heart approaches herald a transformative era for cardiovascular research and precision cardiology. In this perspective, we describe a digital heart ecosystem (Fig. 1) comprising four core micro-ecosystems: devices and data, digital tools, digital discovery and precision cardiology, and infrastructure and governance. These components are interlinked through iterative data and knowledge flows: data from devices inform digital tools for patient-specific modeling, enabling digital discovery and hypothesis testing; the resulting insights guide clinical decision-making and are refined through feedback, supported by enabling infrastructure and governance. These interactions occur both within and across micro-ecosystems, forming a tightly coupled, continuously evolving system. We hope to catalyze discussion that advances the ecosystem and ultimately improve cardiovascular outcomes.

**Figure 1.**
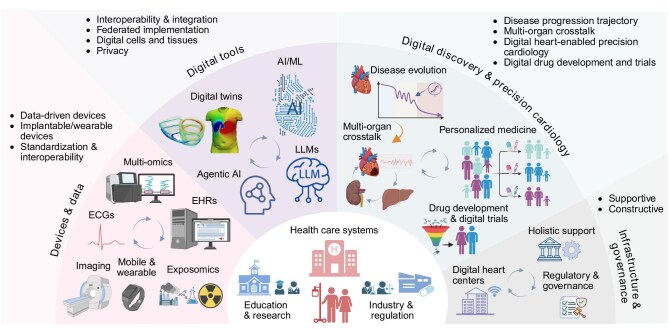
Conceptual framework of the digital heart ecosystem. The proposed digital heart ecosystem consists of four primary micro-ecosystems, namely devices and data, digital tools, digital discovery and precision cardiology, and infrastructure and governance. ECG, electrocardiogram; EHR, electronic health record; AI, artificial intelligence; ML, machine learning; and LLM, large language model. Created in BioRender, https://BioRender.com/754ecyo. A graphical element of digital twins was adapted from Ref. [16] under CC BY 4.0.

### Devices and data

The diagnosis and monitoring of cardiovascular conditions rely on data spanning clinical imaging, electrophysiology, genomics, and wearable sensors [2]. To promote synergistic innovation in medical devices and data analytics, we advocate a unified micro-ecosystem integrating devices and data. Building this device-data nexus requires two priorities: data-driven device innovation and data structuring and analytics. First, although medical device innovation has traditionally been hardware-centric and remains dependent on materials and engineering advances, it should be guided by data-driven methods that optimize design, implementation, and clinical use. For example, clinical data can calibrate cuffless blood pressure monitoring across patients; multi-omics and cohort data can inform imaging probe development for acute coronary syndrome risk assessment and heart failure stratification; and clinical data can guide individualized MRI parameter optimization. A strong device-data nexus may enable personalized, value-based diagnostics, by prioritizing high-value modalities and minimizing low-value tests. Second, data structuring and analytics must address cleaning, standardization, integration, and federated access. Cardiovascular data are heterogeneous across modalities, devices, parameters, and standards, creating substantial inconsistencies. Fragmentation is compounded by barriers between hospitals and patients’ use of multiple healthcare systems, hindering cohesive longitudinal records. Unified data standards and federated access protocols are therefore essential for interoperability across sources and sites. Analytics should integrate clinical data with continuous monitoring from wearable and mobile technologies [3] and environmental exposomics [4], enabling more precise construction and updating of ‘digital patients’ and laying the foundation for personalized cardiovascular medicine.

## Digital tools

Digital tools are central to the digital heart ecosystem and promise to enable data-driven approaches for precision cardiovascular medicine. Key emerging tools include digital twins, conventional artificial intelligence (AI) and machine learning (ML), and large language models (LLMs), as introduced below.

A cardiac digital twin (CDT) is a computational model of a patient’s heart, or part of it, with bidirectional connections between the virtual and physical hearts, enabling dynamic updates, calibration, and targeted predictions over time [5]. CDTs are built on biophysically detailed models of cardiac physiology, tissue and fluid mechanics, derived from mathematical synthesis of (patho)physiological principles, and personalized by assimilating patient-specific clinical data to create a personalized virtual heart replica with mechanistic insights. Recent advances use patient-specific virtual hearts to personalize catheter ablation planning for arrhythmias, including atrial fibrillation and ventricular tachycardia [6], and to reveal sex-specific mechanisms in ventricular conduction and repolarization at the population level [7]. Coronary digital twins integrating coronary computed tomography angiography with computational hemodynamics enable personalized quantification of coronary flow reserve to guide diagnosis and management of coronary artery disease [8].

AI/ML tools have substantial diagnostic and prognostic capabilities for cardiovascular diseases [2]. Studies have applied classic algorithms, such as support vector machines, and deep learning (for example, convolutional neural network) algorithms to electrocardiograms and cardiovascular images for intelligent diagnosis [2], both within or beyond their conventional diagnostic domains.

LLMs are transformer-based generative AI systems trained to produce human-like text and characterized by vast parameter counts. They are versatile in analyzing electronic health records and support complex cardiology care [9], while specialized LLM-based ‘digital cell’ models trained on genomics and transcriptomics data [10] can accelerate computational biology research, emerging as disruptive digital tools for cardiovascular discovery.

Integrating these digital tools into cardiovascular research and clinical practice requires technological breakthroughs and coordinated strategies to establish reliability and adoption pathways. This includes rigorous standards for assessing physiological robustness and trustworthiness, while refining personalization with patient-specific data and emerging inputs from wearables and exposomics. For AI/ML and LLMs, realizing the transformative potential in cardiovascular medicine depends on continuous improvements in real-world performance and explainability. For LLM-based digital cell models, this means curating standardized cardiovascular-specific datasets with perturbation responses and architectures capable of integrating multi-omics data.

Importantly, complementary modeling paradigms should be integrated across digital-tool classes through modular integration and federated interoperability, enabling models to operate across distributed sites. For instance, automated CDT pipelines can use AI/ML to accelerate model construction and personalization via imaging-based processing, enable rapid model evolution within clinically relevant time windows through surrogate-backed parameterization and sensitivity analysis [7], and couple with other site-based tools through federated systems. LLM-based agentic technologies are also emerging to support expert-level application and increasingly automated management of these tools. In parallel, LLM-enabled and ordinary differential equation (ODE)-based digital cells offer significant opportunities for integration. LLM-enabled digital cells, trained on large-scale transcriptomic data, capture transcriptomic states and regulatory programs, whereas ODE-based models simulate cellular electrophysiology, calcium handling, and contractile dynamics through biophysical formulations. Coupling these approaches allows LLM-inferred molecular states to parameterize ODE components (for example, channel conductances or transporter kinetics), while ODE-derived outputs (for example, action potentials and calcium transients) provide mechanistic constraints and synthetic phenotypic data to guide LLM training. This bidirectional integration supports multiscale modeling from gene regulation to cellular function, improving interpretability and predictive capability in cardiovascular diseases.

## Digital discovery and precision cardiology

The digital heart approaches constitute unique and outstanding opportunity to tackle significant challenges in cardiovascular discovery and empower precision cardiology.

First, digital discovery can prioritize mapping the spatiotemporal progression of cardiovascular diseases, advancing understanding of their pathological trajectories. Although not yet realized, this direction is motivated by advances in multimodal longitudinal patient cohorts and emerging cardiac organoid platforms that enable human-oriented interrogation of disease progression at high spatiotemporal resolution, while digital approaches could systematically reconstruct and interpret disease trajectories. In contrast, existing cardiovascular studies often rely on animal models or diseased human heart samples, creating ‘snapshots’ frequently captured after disease initiation. Concerted digital cell and tissue models can recapitulate cardiovascular pathogenesis and progression, providing continuous, mechanistic insights into temporal disease evolution as ‘movies.’ These digital tools should also untangle interactive networks spanning from molecular underpinnings of intercellular communication to inter-organ crosstalk [11]. Because cardiovascular diseases often involve other organs and dysfunctions in peripheral and intra-cardiac nervous systems, multi-organ digital tools with spatiotemporal pathology details could reveal early digital biomarkers for diagnosis and prevention. Such integrative frameworks promise to accelerate targeted therapy development through digital clinical trials.

Second, precision medicine requires proactive, personalized intervention optimization, demanding rapid, robust, and accurate treatment-outcome predictions. By simulating personalized therapies through predictive modeling, digital heart technologies are uniquely positioned to advance precision cardiology, addressing the urgent need for supporting individualized therapeutic strategies and decision making for major cardiovascular diseases such as heart failure, atrial fibrillation, and acute coronary syndromes. Integrated with mobile and wearable technologies, these models can further enable effective telemedicine for chronic heart conditions. Moreover, digital tools such as LLMs and CDTs could transform the current paradigm for patient–clinician encounters, as well as medical education. Ultimately, the effectiveness and impact of these technologies must be validated through prospective clinical trials.

Third, digital clinical trials, powered by AI-generated synthetic patient data and CDT-based virtual cohorts, offer a compelling extension to conventional randomized controlled trials by enabling controlled, reproducible exploration of expanded intervention and parameter spaces, while reducing cost, logistical burden, patient risk, and ethical constraints [12]. Rather than replacing traditional trials, they can enhance trial design through *in silico* hypothesis testing, cohort stratification, and early narrowing of viable interventions, supporting smaller, targeted studies [13]. Emerging agentic systems may further advance this paradigm through iterative, model-informed trial design and execution with continuous patient-level engagement. Virtual CDT cohorts integrating real-world data streams could enable adaptive refinement of inclusion criteria, endpoint selection, and intervention strategies. Together, these advances point toward integrated digital–clinical ecosystems for more responsive, patient-centered, and precise cardiovascular trials.

## Infrastructure and governance

To consolidate foundational pillars of a thriving digital heart ecosystem, major investment and supportive governance are urgently needed. This includes specialized cardiovascular data centers and federated infrastructure to securely host and apply digital tools, alongside regulatory frameworks that safeguard high standards for data, tools, privacy, and clinical accountability. Holistic support for digital heart research and education can be advanced through dedicated academic programs and professional societies.

## Toward longitudinal and agentic CDTs

Together, these four microecosystems define the foundation of a digital heart ecosystem; their convergence could transform CDTs from static, snapshot-based models into longitudinal, adaptive systems supported by agentic frameworks [14].

Realizing this transition will require advances beyond current CDTs. First, increasing biological fidelity is a priority. Incorporating additional layers of biological complexity, such as inflammation, metabolism, and genetic variation, through mechanistic ODE-based modeling or AI/LLM-derived representations, will enable more comprehensive, personalized CDTs by allowing direct assimilation of diverse, high-dimensional data. Second, advances in computational technologies are essential for clinical translation. AI-based surrogate modeling [15] can substantially reduce the cost of high-fidelity simulations and facilitate model evolution, enabling routine uncertainty quantification that improves reliability and interpretability for clinical decision-making.

Powered by these advances, longitudinal CDTs can be derived from longitudinal data. Current CDTs rely on snapshot data and are limited to acute prediction. Future CDTs are expected to become continuously updating systems that integrate imaging, EHRs, wearable, and exposomic data, enabling closed-loop learning and progressively refined patient-specific modeling. This shift may allow representation of disease progression through structural, metabolic, and inflammatory remodeling, improve prediction of treatment responses, optimize interventions such as cardiac ablation and cardiac resynchronization therapy, and extend prediction to long-term physiological trajectories.

Lastly, we propose an agentic CDT framework—a hybrid system in which agentic workflows augment mechanistic CDTs and integrate generative models of cardiac remodeling. Acting as scientific assistants under validated protocols, agentic layers orchestrate the end-to-end pipeline, including data quality control, multimodal alignment, model selection, calibration, and simulation. They facilitate hypothesis generation, recommend modeling strategies, enforce reproducibility, and support governance across heterogeneous components (for example, selecting context-appropriate formulations). Alongside this orchestration layer, a generative remodeling layer trained on longitudinal cohorts captures slower structural, electrical, and metabolic adaptations. The functional (that is, CDTs) and remodeling layers are bidirectionally linked: phenotypic outcomes from the functional layer inform remodeling trajectories, which update the CDT functional substrates, enabling prediction of both acute responses and long-term outcomes, including atherosclerotic plaque progression and post-intervention remodeling. In this form, agentic CDTs become clinically actionable infrastructure for precision cardiology.

In summary, the digital heart ecosystem promises to transform cardiovascular discovery and care, paving a realistic path toward precision cardiology. Realizing this vision will require coordinated stakeholder support and sustained R&D to address key challenges. The ecosystem must remain inclusive, interoperable, and readily integrated with other organ-specific platforms and broader digital human frameworks. National centers, academic consortia, and dedicated conferences will be vital for catalyzing progress, while thoughtful governance will ensure responsible, sustainable evolution. Ultimately, the digital heart ecosystem will redefine how cardiovascular diseases are studied, diagnosed, and treated.
